# Outer membrane protein C (OMPC) epitope of *Shigella flexneri* 3a as a potential marker of primary immunodeficiencies (PID) and isolated anti-OmpC antibodies as a tool for immunoglobulin replacement therapy

**DOI:** 10.3389/fimmu.2025.1701211

**Published:** 2025-11-19

**Authors:** Piotr Naporowski, Danuta Witkowska, Jacek Rybka, Edyta Pawlak, Aleksandra Lewandowicz-Uszyńska, Ewa Masłowska, Andrzej Gamian

**Affiliations:** 1 Hirszfeld Institute of Immunology and Experimental Therapy, Polish Academy of Sciences, Wroclaw, Poland; 2 3rd Department and Clinic of Paediatrics, Immunology and Rheumatology of Developmental Age, Wroclaw Medical University, Wroclaw, Poland; 3 Department of Immunology and Paediatrics, J. Gromkowski Regional Specialist Hospital in Wroclaw, Wroclaw, Poland; 4 1st Department of Paediatrics, Alergology and Cardiology, Wroclaw Medical University, Wroclaw, Poland

**Keywords:** *Shigella*, OmpC, primary immunodeficiency (PID), diagnostic marker, antibody supplementation

## Abstract

**Introduction:**

Over 6 million people worldwide are affected by primary immunodeficiencies (PIDs), which often remain undiagnosed, and the diagnostic process is complex and challenging. Dysfunction of the immune system can lead to permanent damage to body systems and organs; moreover, Ig replacement therapy carries the risk of anaphylactic shock following the administration of the immunoglobulin preparation. The present study proposes an alternative testing method for IgA deficiency, using the BSA-peptide conjugate with the RYDERY sequence, which may serve as a simpler alternative to complex diagnostic schemes.

**Methods:**

We analysed the levels of anti-OmpC *S. flexneri* 3a antibodies in sera from healthy individuals (40 samples from children and 66 samples from adult blood donors) and patients (127 samples from patients with PID, 83 samples from patients with RRTI), utilising the native bacterial OmpC protein and two BSA-peptide conjugates: one linear and one with a cyclic structure.

**Results:**

The obtained results showed that for OmpC and both conjugates, IgA titres – unlike IgG – were significantly lower in patients with PID and RRTI compared to healthy controls. Additionally, the levels of specific IgA antibodies differed significantly between men and women in both the PID patient and healthy adult groups when using native OmpC protein, but not when employing conjugates as the antigen. These findings strongly support using the conjugate, particularly with the linear peptide, instead of the whole OmpC protein in immunochemical assays. The level of IgA in patients’ sera is generally lower compared to that of healthy controls and decreases with age when conjugates are used for analysis. In the mouse model, specific, isolated anti-OmpC antibodies from both human and mouse serum had similar protective activity against *Shigella* infection.

**Discussion:**

The results demonstrate that the additional use of the cyclic/linear peptide-BSA conjugate offers a significant advantage over the use of the complete OmpC protein for immunological testing in PID diagnostics. Furthermore, specific anti-OmpC antibodies may be beneficial in the complementary therapy for patients with PIDS.

## Introduction

Gram-negative bacteria of the *Enterobacteriaceae* family are etiological factors of diarrhoea, typhoid fever, bacterial dysentery, and other intestinal diseases. These pathogens are a serious concern, especially in developing countries, where they are responsible for a high number of fatalities among patients (1). Infection typically occurs through the ingestion of contaminated food and water or through direct contact, resulting in acute gastroenteritis. Characteristic symptoms of shigellosis include watery diarrhoea with blood or mucus, nausea and vomiting, high fever, and abdominal pain (2). Particularly dangerous shigellosis (bacillary dysentery) occurs in children under 5 years of age, seniors, and people with impaired immune system functions. Resistance to antibiotics and other antimicrobial drugs also poses a significant challenge to the effective treatment of bacterial infections.

Immunodeficiency is a condition of the body in which some of the functions of the immune system are impaired or do not work at all. Primary immunodeficiencies (PIDs) are a heterogeneous group of genetically determined diseases resulting from disorders of the structure, maturation, differentiation, and function of cells and proteins within the immune system. While secondary immunodeficiency is more common than primary (or congenital) immunodeficiency, up to 90% of cases remain undiagnosed (3). PIDs, as rare diseases often associated with antibody deficiency, occur at a frequency of one in 300 to 500 live births. Due to the rarity of these conditions, their diagnosis is often delayed or not diagnosed until adulthood. PIDs increase the vulnerability to infection, which can lead to chronic conditions and generate long-term healthcare needs. Due to such congenital defects of the immune system, infections develop more rapidly, are more challenging to treat and frequently recur (1, 4). PIDs can also predispose to cancer and autoimmune diseases (3). The most effective, least invasive and safest way to fight pathogens is treatment with intravenous immunoglobulin preparations (IVIG – intravenous immunoglobulins) or with immunoglobulins in the form of subcutaneous injections (5, 6). Prevention and treatment of infections by supplementing antibody deficiencies is a very promising therapy for many patients, including those suffering from diseases associated with PID and SID.

In patients with PIDs, the most common manifestations of bacterial infections include chronic sinusitis, pneumonia, and otitis media. Patients with diagnosed IgA deficiency often experience recurrent respiratory infections (7).

The etiological factors of infections in patients with PID are often bacteria from the *Enterobacteriaceae* family, mainly of the genus *Shigella*. *Shigella flexneri* and *Shigella sonnei*, which cause shigellosis, also known as bacterial dysentery or colitis. In patients with deficiencies, they cannot be treated entirely and often recur, causing severe inflammation of the rectal and intestinal mucosa, accompanied by bloody and purulent diarrhoea (4, 8). In the case of these patients, we can diagnose the deficiency in antibody production before the onset of infection and prevent infection by immunoglobulin supplementation, or facilitate treatment once it has occurred.

Outer membrane proteins are part of the cell envelope of Gram-negative bacteria and participate in maintaining cell integrity, pathogen adaptation to the environment and interaction with the host cells (12). Outer membrane protein C (OmpC) is a one of the major porins from OMP group and has been shown to be highly immunogenic antigens, efficiently eliciting protective antibodies and have a high degree of sequence and structure homology (42). The 39 kDa protein from *S. flexnerii* 3a, consisting of 352 amino acids (the determined sequence: Gene Bank 24113600), is composed of three beta-barrels acting as a channel for nutrient and water transport. This protein is crucial for bacterial survival and is involved in pathogenesis, with its channels regulated by environmental factors like osmolarity and antibiotics. The OMPs markedly contribute to the mechanisms of pathogenicity, progression of the infection, and to the development of the inflammatory response (12). Our previous results shown, that from five potential epitopes predicted from the three-dimensional model of OmpC from *S. flexnerii* 3a only one, i.e. loop V, was recognized by human sera (15). Peptide mapping of the loop V narrowed down the epitope’s sequence to RYDERY. The same epitope was preferentially recognized by mice sera accounting for approx. 70% of OmpC’s antigenic activity (15).

Our earlier study (2) showed that 39 kDa outer membrane protein C (OmpC) isolated from *S. flexneri* 3a is highly immunogenic and plays a central protective role among other outer membrane proteins in the *Enterobacteriaceae* family. In that work, we have demonstrated that active immunisation of mice protected them against infection with live pathogens (2, 9). In serological studies, we have shown that OmpC reacts not only with serum from immunised mice but also with normal human serum and human umbilical cord plasma with high affinity (19). Additionally, we observed anti-OmpC antibodies in the sera of PID patients (both children and adults), but at significantly lower levels than in the sera of healthy individuals (2, 10). The anti-OmpC antibodies present in the umbilical cord blood are transferred to the baby, which may indicate that they may have a protective function. Mapping the epitope recognized by anti-OmpC antibodies enabled us to determine its 12-amino acid sequence (GLNRYDERYIGC) and the shortest sequence of this peptide (RYDERY), which is necessary for effective interaction with anti-OmpC antibodies present in the serum (11).

Our previous results (12) have inspired us to further study the level of anti-OmpC antibodies in sera obtained from a large group of patients (children and adults) with PID, patients with recurrent inflammation of the upper respiratory tract and healthy donors, as they indicated that the OmpC protein can serve as a potential marker of PID and the specific epitope of this protein can be used as an antibody-binding antigen. Such protective antibodies can be used in the future for the treatment of patients with PID, which could be of great importance, as the wide range of PID presentations results in some immunodeficiency forms remaining undiagnosed for years (3).

The primary objective of the present work was to determine the level of specific anti-OmpC *S. flexneri* 3a antibodies in a larger number of sera from healthy individuals and immunocompromised patients, and to verify whether synthetic peptides containing the RYDERY sequence can be more useful for PID testing purposes. Additionally, the study aimed to investigate the protective properties of anti-OmpC antibodies isolated from human or mouse peripheral blood in an animal model. These antibodies are primarily directed against epitopes containing the RYDERY sequence and will be referred to as anti-RYDERY antibodies throughout the text. As primary immunodeficiencies continue to increase in prevalence as a medical problem, discovering novel diagnostic tool and therapeutic method will be crucial for a better understanding of disease patterns and improved patient treatment.

## Materials and methods

### Bacterial strain and culture condition

*S. flexneri* serotype 3a strain PCM 1793 from the Polish Collection of Microorganisms (PCM) of the Institute of Immunology and Experimental Therapy, Polish Academy of Sciences, was used in the study. Bacteria were grown on enriched Bacto Agar plates (Difco) and cultured for 8 hours with gentle shaking in liquid Brain-Heart Infusion (BHI) medium (Difco).

### Sera

Human and mouse sera were used in this study. Blood donors’ sera (66 samples in total) were obtained from the Military Centre for Blood Donation, Local Branch Station in Wroclaw. Serum samples from healthy children (40 samples) and patients with diagnosed PID (PID – primary immunodeficiency diseases) (127 samples) as well as RRTI patients (RRTI - recurrent respiratory tract infections) (83 samples) were obtained from 3^rd^ Department and Clinic of Paediatrics, Immunology and Rheumatology of Developmental Age, Wroclaw Medical University, Poland; Department of Immunology and Paediatrics, J. Gromkowski Regional Specialist Hospital in Wroclaw; Poland and Department of Paediatrics and Alergology and Cardiology Wroclaw Medical University. All serum samples were collected from patients from Wroclaw and the Lower Silesia region. All samples were anonimized. Samples were collected over relatively long time, often was given in a very small volume, and were not always accompanied with complete information about the patient gender and age. Therefore the number of samples used differed in experiments. The exact number of samples used in the particular experiment is presented in the respective table.

The study was approved by the Medical Ethics Committee of the Medical University of Wroclaw (No KB-144/2020), and all experiments were performed in accordance with its guidelines and regulations. The study on the animal model was approved by the Local Ethical Commission for Animal Experiments (No. 020/2021/P1). In the experiment, where humoral protective activity against the LD_50_ dose of bacteria was tested, serum samples from mice immunised thrice with the OmpC (50 µg doses), the preparation of which was described in a previous publication (2), were used.

### Animals and animal model to study the protective properties of specific antibodies

All experiments were performed on six to seven-week-old female BALB/c mice (20–25 g) obtained from the Mossakowski Institute of Experimental and Clinical Medicine of the Polish Academy of Sciences in Warsaw, Poland, and randomly divided into four groups of 10 animals each. Mice were placed in cages enriched with toys and nesting material, with constant access to water and standard feed. The parameters in the animal rooms were set to: temperature, 22 ± 2°C; humidity, 55% ± 10%; and lighting, 12/12 hours. Before the experiments, the animals were quarantined for one week. Animal care staff responsible for mice immunisation were unaware of the allocation group to ensure that all animals in the experiment were handled, monitored and treated in the same way. The table below (**Table 1**) contains a detailed protocol for testing the protective activity of human/mouse antibodies against the OMPC protein from *S. flexneri* 3a.

**Table 1:** Scheme of passive immunisation (200 µl intraperitoneally) of mice using specific anti-OmpC antibodies obtained from human or mouse serum resolved in 200 µl saline, applied in an experiment with infecting dose of LD_50_*S. flexneri* 3a (7x10^7^ cells/mouse).

No of group	Day 1	Day 2	Day 3
1	PBS control group	PBS	PBS control group
		1 hour interval	
		LD_50_*Shigella flexneri*	
2	50 µg Abs human	50 µg Abs human	50 µg Abs human
		1 hour interval	
		LD_50_*Shigella flexneri*	
3	100 µg Abs human	100 µg Abs human	100 µg Abs human
		1 hour interval	
		LD_50_*Shigella flexneri*	
4	50 µg Abs mouse	50 µg Abs mouse	50 µg Abs mouse
		1 hour interval	
		LD_50_*Shigella flexneri*	

The control group of mice was intraperitoneally treated with 200 µl of saline instead of antibodies. The experiments were designed in accordance with the guidelines of the National Ethics Committee and approved by the First Local Ethics Committee at the Institute of Immunology and Experimental Therapy of the Polish Academy of Sciences (LKE 020/2021/P1, 21 April 2021).

### Isolation of bacterial outer membrane proteins

Bacteria were grown at 37°C in liquid BHI (Brain Heart Infusion, Difco) medium for 8 hours and harvested by centrifugation. A crude outer membrane protein (OMPs) fraction was extracted from dry *S. flexneri* 3a bacterial mass with valeric acid according to Arcidiacono et al. (13). The OmpC from the crude membrane fraction was purified by gel filtration and ion-exchange chromatography, as outlined in the following protocol. Extracted proteins were suspended in a buffer containing 1% Triton X-100, 50 mM Tris-HCl (pH 8), and 50 mM NaCl. The suspension was then centrifuged at 14,000 × g for 30 min. The supernatant containing OMPs was loaded onto a column (1.6×100 cm) with Sephacryl S-200 HR (Pharmacia), which was equilibrated with a 0.4% Triton X-100 solution in a 50 mM Tris-HCl buffer (pH 8) and 50 mM NaCl. Purification of the proteins from the extract was carried out using an FPLC system at a flow rate of 0.3 ml/min in the isocratic mode, collecting 2 ml fractions. Fractions containing OmpC were pooled, dialysed against water, and concentrated by ultrafiltration (30-kDa cut-off membrane, Millipore).

The concentrated sample was loaded onto an ion-exchange DE-52 HR (Whatman) column (1.6 cm × 10 cm) that was equilibrated with a buffer containing 50 mM Tris-HCl, 50 mM NaCl, and 0.4% Triton X-100, at pH 8.0. The OmpC was eluted with a linear gradient up to 0.5 M NaCl at a flow rate of 0.3 ml/min. OmpC fractions, as determined by SDS-PAGE electrophoresis, were pooled, dialysed into water, and concentrated using ultrafiltration on Amicon (10 kDa cut off, Millipore). Protein concentration was determined using the Bradford method (Thermo Fisher Scientific), with BSA as the standard.

### Synthetic peptides

The linear peptide with the sequence GLNRYDERYIGC was obtained from NOVAZYM POLSKA s.c. According to the product quality certificate, the peptide was obtained through organic synthesis. The determined *m/z* value of the peptide was 1458.00 kDa, which corresponds to a theoretical mass of 1458.60 kDa. The cyclic peptide with the sequence [GGLNRYDERYIGK]-C was obtained by organic synthesis thanks to cooperation with the Team of Chemistry and Stereochemistry of Peptides and Proteins, Faculty of Chemistry, University of Wroclaw. Peptide purification was carried out using preparative liquid chromatography on a Varian ProStar apparatus (column: TSKgel ODS-120T 12TG08eh004 + Guard Column, UV detector (λ = 210 nm and 280 nm), injection volume: 2 ml, flow: 7 ml/min). Eluted with solution A (H_2_O + 0.1% TFA) and B (80% CH_3_CN/H_2_O + 0.1% TFA) in a gradient of solution B in the range of 0–80% for 40 minutes. 10 mg of peptide was obtained from 200 mM TCEP (TCEP – tris (2-carboxyethyl) phosphine) in lyophilised form. Then it was subjected to ESI-MS mass analysis, performed on microTOF-Q (Bruker Daltonics, Bremen, Germany) and Apex Ultra FT-ICR 7T (Bruker Daltonics, Bremen, Germany) mass spectrometers with an ESI ion source (ESI - Electrospray Ionisation) in positive ion mode. The instrument was calibrated using TuneMix (Agilent Technologies, Santa Clara, USA). A CH_3_CN/H_2_O/HCOOH solution (50:50:0.1 v/v/v) was used for measurements. The measured *m/z* value was 904.934, which corresponds to the theoretical value of the [M + 2H]^2+^ ion of 904.922.

### Peptide conjugation with BSA as a carrier

BSA monomer was purified by gel filtration of 100 mg BSA (Sigma-Aldrich) dissolved in 1 ml of 0.1 M ammonium acetate on a Sephacryl S-200 bed (GE Healthcare Life Sciences) in the Äkta Explorer FPLC system in 0.1 M ammonium acetate, at a flow rate of 0.3 ml/min, collecting 2 ml fractions. Fractions containing the BSA monomer were pooled, dialysed against water, and lyophilised to yield 36 mg of monomer BSA. The BSA monomer was then used for the conjugation of peptides. Before conjugation, free amine groups on the carrier protein (BSA) were bromoacetylated in 0.1 M carbonate buffer, pH 8.3, using 1 mg N-hydroxysuccinimide bromoacetic acid ester per 1mg of BSA (0.5 ml). The sample was bromoacetylated for 3 hours at room temperature, maintaining a constant pH of 8.3 with 0.1 M NaOH on a rotary mixer. The reaction was stopped by rinsing the sample with 0.1 M carbonate buffer on a 30-kDa Centricone (Milipore). The protein sample was concentrated to 10–20 mg/ml, and the bromoacetylation efficiency was confirmed by determining the percentage of remaining free amino groups on BSA using the TNBS method (13). Peptides containing C-terminal cysteine were dissolved in a 10-fold excess of methylphosphine and dried under argon in tightly closed vials. The peptides were resuspended in 0.1 M carbonate buffer with 2 mM EDTA, pH 8.3, to a final concentration of 10–50 mg/ml. Peptides were conjugated with bromoacetylated BSA (2 mg/ml) at a ratio of 1:50-100 (mol:mol) at pH 8.5. The conjugation reaction was carried out for 16 hours at room temperature in a rotary shaker. The free bromoacetylated groups were inactivated with 140 mM 2-mercaptoethanol for 1 h at room temperature (14). The degree of BSA substitution with peptides was assessed by MALDI-TOF-MS.

### Enzyme-linked immunosorbent assay

The immunoreactivity of the antigens: native OmpC (0.5 µg/well), conjugated BSA with linear peptide (GLNRYDERYIGC: BSA (0.167 µg/well) or with cyclic peptide (GLNRYDERYIGK)-C:BSA (0.250 µg/well) with human sera IgA and IgG was measured by ELISA. The experiment was performed as described in detail (12, 15). In short, each well of the 96-well plates (MaxiSorp, Thermo Fisher Scientific) was coated with 0.1 ml of antigen in coating buffer, pH 9.6, at 4°C overnight. Control wells were treated with coating buffer only. Unadsorbed antigen was removed by washing the wells three times with 200 μl of TBS-T buffer. The plate was blocked with 1% BSA (Kierkegaard & Perry Laboratories) in TBS-T buffer, pH 7.5, at room temperature for 1 hour. After blocking, all wells were washed three times with 200 μl TBS-T. The wells were then filled with 100 µl/well of human sera (diluted with TBS-T 1:750 for IgG test and 1:250 for IgA test) and incubated at room temperature for 2 hours. After washing three times with TBS-T, 100 µl/well of an alkaline phosphatase-labelled goat anti-human IgG diluted 1:10000 or an alkaline phosphatase-labelled goat anti-human IgA diluted 1:20000 was added to each well. Following a 1-hour incubation at room temperature, the plates were washed with 200 μl of TBS-T and then incubated with 150 μl of APYellow (pNPP) substrate (Sigma). After 30 min, the reaction was stopped by adding 50 μl of 3 M NaOH, and the optical density was read at 405 nm using an automatic microplate reader (BioTek).

### SDS-PAGE and immunoblotting

The OmpC samples were characterised by SDS-PAGE, as described by Laemmli (16), using 12.5% gels on the Biometra system. Protein molecular mass estimation was performed using the LMW Calibration Kit for SDS Electrophoresis, Amersham, GE Healthcare.

Separated proteins were transferred to an Immobilon P membrane (Millipore) using the Trans-blot system (Bio-Rad, Richmond, CA) with transfer buffer (10 mM Tris, 150 mM glycine, 20% methanol) at 4°C for 1 h at 100 V (12, 15). After 1 hour blocking with 1% BSA in TBS-T, the membrane with transferred proteins was incubated for 1 hour at 37°C with human serum diluted 1:100 in 1% BSA in TBS-T (20 mM Tris-HCl, 50 mM NaCl, 0.05% Tween 20, pH 7.5). Thereafter, the membrane was incubated for 1 hour at 37°C with goat anti-human IgG antibodies conjugated with alkaline phosphatase diluted to 1:10,000 in TBS-T. After washing the membrane three times in TBS-T, immunoblots were developed with substrate for alkaline phosphatase (NBT/BCIP) in 100 mM Tris-HCl, pH 9.5, containing 100 mM NaCl and 50 mM MgCl_2_.

### Determination of free amino groups on the protein surface

A protein solution in the concentration range of 30 – 80 µg/ml with a volume of 50 µl or a standard solution (glycine) in the concentration range of 0 – 12 µg/ml and 50 µl of a 0.1 M carbonate buffer solution with a pH of 8.5 were incubated for 2 hours with 50 µl 0.02% TNBSA (2,4,6-trinitrobenzenesulfonic acid) at 37°C. The reaction was stopped by adding 25 μl of 10% sodium dodecyl sulfate (SDS) and 15 μl of 1 M hydrochloric acid (HCl). The absorbance was measured at a wavelength of λ = 335 nm (13).

### MALDI-TOF-MS analysis

The analysis of BSA and BSA-cyclic/linear peptide conjugates was performed using the MALDI-TOF-MS technique, using as a matrix a 20 mg/ml solution of α-cyano-4-hydroxycinnamic acid (HCCA) in FWI: (formic acid/water/isopropanol in a ratio of 3/1/2 v/v/v). On a spot of a steel target plate, 1 µl of matrix was mixed with 1 µl of BSA or a BSA-peptide conjugate and then air-dried. Mass analysis was performed using the ultrafleXtreme apparatus with flexControl software and analysed in the flexAnalysis program (Bruker Daltonics).

### Preparation of the affinity chromatography column

To 2 ml of Sepharose CL-4B (Pharmacia), 2 ml of acetonitrile was added, and the pH was adjusted to 11.0 with a 4 M NaOH solution. After the addition of 0.2 g of CNBr (cyanogen bromide), the gel was incubated at room temperature on a rotary stirrer for 30 minutes under an extractor, while maintaining a pH of 11.0. The activated Sepharose-CNBr was washed on a sinter funnel with 10 ml of H_2_O and 10 ml of 0.1 M carbonate buffer, pH 8.6. Then, 2 ml (c = 2.5 mg/ml) of OmpC protein dissolved in 0.1 M carbonate buffer (pH 8.6) was added, and the mixture was incubated on a rotary stirrer for 3 hours at room temperature and then overnight in a refrigerator. Free -CN groups were blocked by incubation for 1 hour with 8 ml of 1 M ethanolamine, pH 8, at room temperature. The prepared gel was washed on a sintered funnel with 10 ml aliquots of water and PBS (containing Mg^2+^ and Ca^2+^). The sample, suspended in PBS, was applied to the column and washed with PBS buffer.

### Purification of specific anti-OmpC antibodies from human/mouse serum using a column with immobilised OmpC protein

A 5 ml column with a Sepharose CL-4B bed with immobilised OmpC protein was equilibrated with PBS (Mg^2+^, Ca^2+^). Then, human or mouse serum diluted 1:1 with PBS was applied. Material unbound to the column was washed off with PBS, and proteins not specifically bound were released using 1 M NaCl. Sodium chloride was then removed by washing the column with PBS, and specific anti-OmpC antibodies were subsequently eluted using a 3 M potassium thiocyanate solution (KCNS). Fractions containing specific antibodies were combined, concentrated, and then washed with water using an Amicon with a 30 kDa filter membrane (Merck Millipore).

### Determination of the LD_50_

LD_50_ of *S*. *flexneri* 3a strain was calculated using the method of Reed and Muench, as described in our previous publication (15, 17), where groups of mice were injected intraperitoneally with 0.2 ml of 10-fold increasing dilutions of an overnight broth culture bacteria. The procedure was performed on forty 6–9 week-old female mice of the BALB/c strain. Each of the 4 groups of animals consisted of 10 individuals. The animals were immunized 3 times at a 24-hour interval.

### Statistical analysis

Graphs and statistical analysis were performed using GraphPad Prism 5 software. The Shapiro-Wilk test was used to test the normality of the data. The significance of statistical differences in antibody titres was assessed using the Mann-Whitney test or the T-test. The difference was considered significant at P < 0.05. Data represent means ± SD from three technical replicates. An asterisk symbol (*) indicates p < 0.05, two asterisks (**) indicate p < 0.01 and three asterisks (***) indicate p < 0.001. The analysis of antibody levels in the different study groups is presented in box-plots, where Q1 and Q3 represent quartiles indicating that 25% of the subjects are above the Q1 value and that 25% of the subjects are below the Q3 value. The Pearson correlation coefficient (r-Pearson) was used to determine the relationship between patient age and antibody levels. A coefficient value less than zero indicates a decreasing trend, while a positive r-value indicates a co-proportional increase in age and antibody titres. The coefficient in the range of -0.5 to 0.5 indicates a weak correlation, while value in the range of -1.0 to -0.5 and 0.5 to 1.0 indicates a strong correlation.

## Results

### Isolation and purification of *S. flexneri* 3a OmpC

#### Extraction of outer membrane proteins of S. flexneri 3a

A crude outer membrane protein (OMP) fraction was extracted from 27 g of lyophilised *S. flexneri* 3a bacteria mass using valeric acid at a final concentration of 2.3 M, according to Arcidiacono et al. (18). The extraction was carried out twice; the first yielded 120 mg of protein (Extract I) and the second 35 mg (Extract II). As the protein composition in both extracts was similar, with OmpC protein predominant, the two preparations were pooled.

#### Purification of OmpC protein

**Figure 1:**
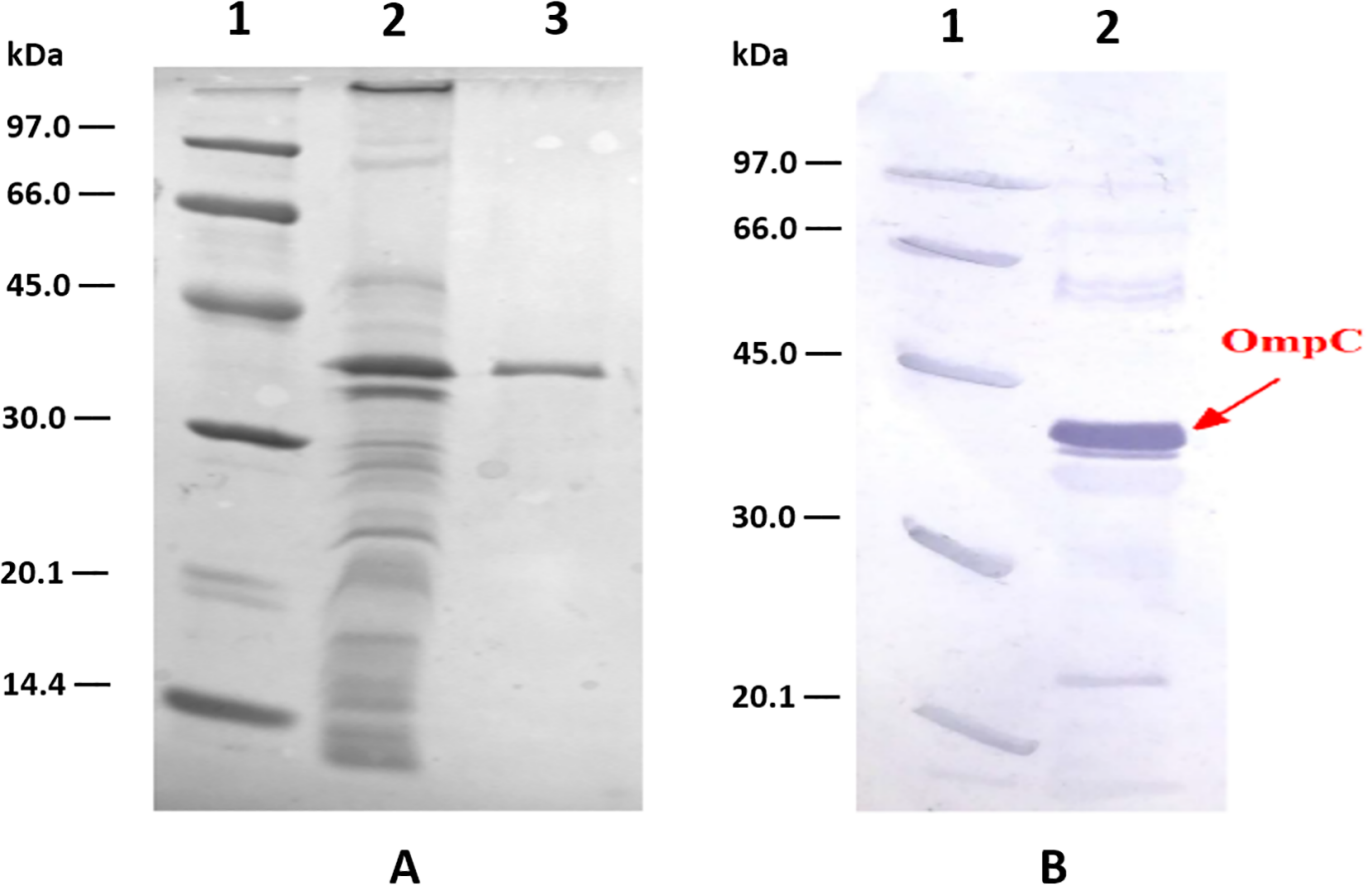
Analysis of OmpC protein preparations from *S. flexneri* 3a: **(A)** – SDS PAGE of: LMW markers, (1) crude outer membrane proteins preparation, (2) and homogeneous OmpC protein sample (5 µg) (3) obtained after ion exchange chromatography on DE-52 column, **(B)** – immunoreactivity of OmpC protein with human serum containing specific anti-OmpC IgG antibody analysed using Western blotting (2), LMW markers (1).

The OmpC was further purified according to the procedure optimised in our laboratory (15). During the process of protein purification, the protein must retain its native structure and natural spatial conformation. This is of considerable importance for further use of the protein in immunoreactivity assays. Therefore, the various purification steps should be short and should not use components that can denature its structure. OmpC is a protein naturally localised in the outer membrane of *S. flexneri* cells. Due to its localisation, there are several hydrophobic amino acids in its primary structure, allowing for its stability and functions in the lipid transmembrane environment. The hydrophobicity of the molecule prevents it from dissolving in aqueous solutions. An essential step in the purification of the post-extraction protein preparation was the selection of a suitable environment. A 0.4% Triton X-100 detergent was used in the chromatography to prevent protein precipitation. In the first step, the gel filtration method was used: the crude Omp fraction (15 mg) obtained by extraction with valeric acid was purified using molecular filtration chromatography on a Sephacryl S-200 column. After gel filtration, the concentrated sample, containing mainly OmpC (5 mg of protein in total), was purified using anion-exchange chromatography. The efficiency of the purification process on the ion exchange column was 36%, and 2.0 mg of OmpC was obtained (**Figure 1**). The overall efficiency of the entire protein purification process was 13.3%.

### Conjugation of BSA monomer with linear and cyclic OmpC epitope peptide

**Figure 2:**
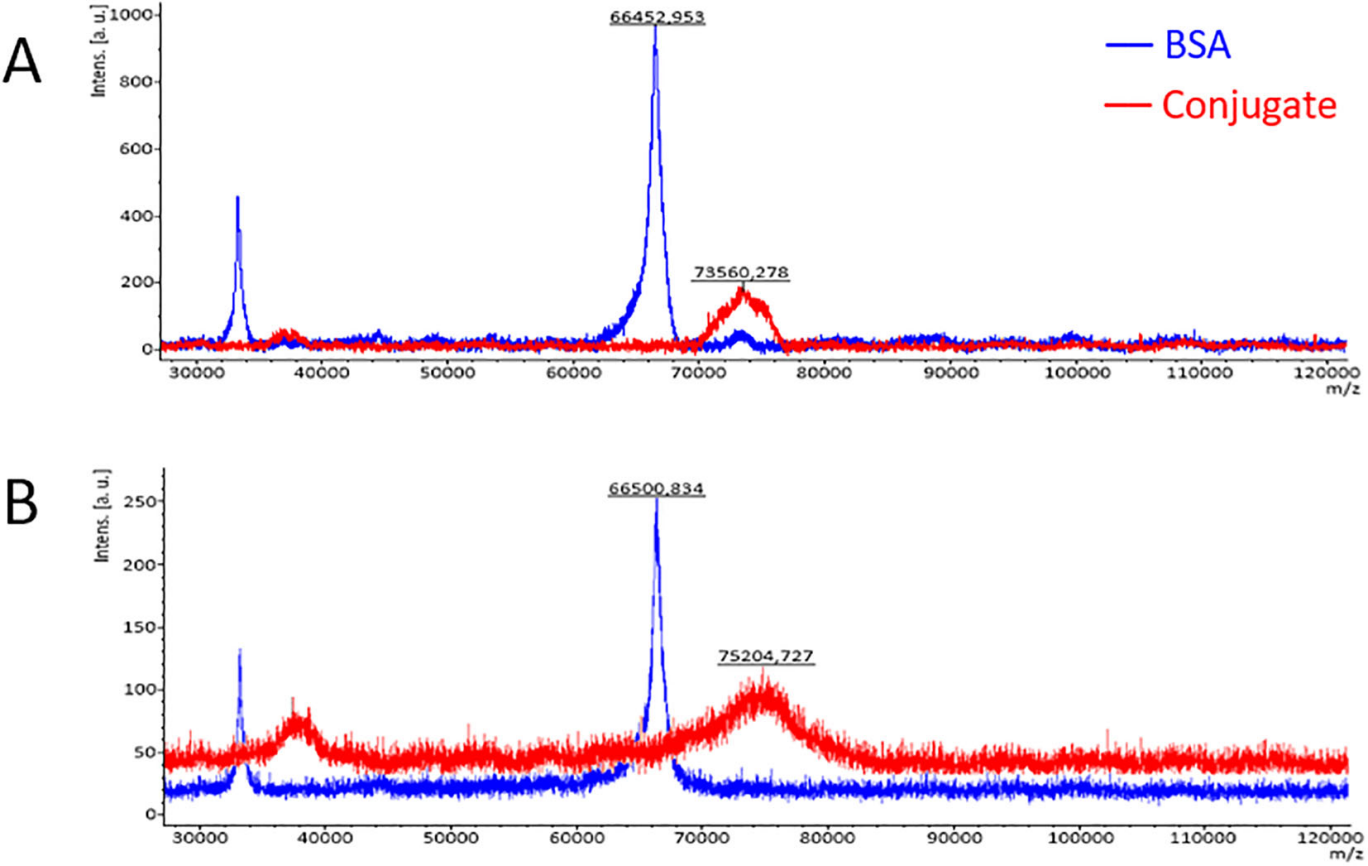
Mass spectra of BSA and the BSA cyclic-peptide conjugate **(A)** and the BSA linear-peptide conjugate **(B)** obtained by the MALDI-TOF-MS technique.

We have used peptides containing the RYDERY sequence in two spatial conformations (linear: GLNRYDERYIGC and cyclic: [GGLNRYDERYIGK]-C) for conjugation with the BSA monomer to obtain conjugates for further studies. The conjugation was performed using the method of binding peptides to bromoacetylated amino groups of the protein. The degree of loading of the carrier protein (BSA) with peptides was determined by the MALDI-TOF-MS technique. The number of attached peptide molecules to BSA was determined from the quotient of the difference between the weight of the conjugate and the weight of BSA and the weight of the peptide (**Figure 2**) according to the formula: peptide occupancy = (M_conjugate_ – M_BSA_)/M_peptide_.

The average occupancy of the carrier protein (BSA) with peptides was calculated to be around 5.97 for the BSA/linear peptide conjugate and 3.93 for BSA/cyclic peptide, which corresponds to the average of 4 molecules of cyclic peptide and 6 molecules of linear peptide per BSA molecule. The peaks of the mass spectra of the conjugates on the mass spectrum (red in **Figure 2**) are bell-shaped, indicating variable degree of peptide occupancy of the carrier protein, what may be due to their spatial conformation.

### Isolation of specific human or mouse anti-OmpC antibodies using affinity chromatography on a Sepharose CL-4B column with immobilised OmpC protein

**Figure 3:**
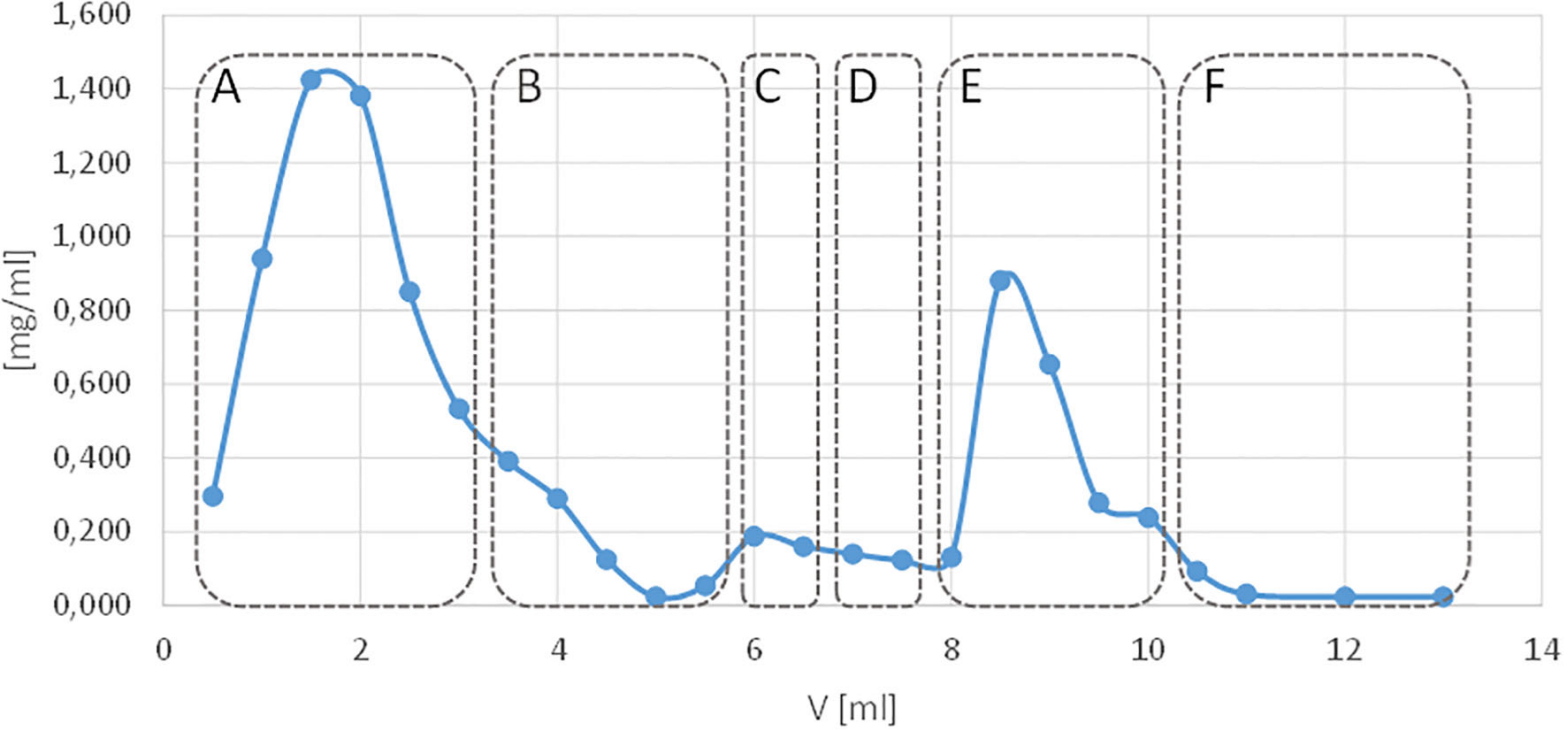
Elution profile of human peripheral blood serum antibodies from a Sepharose CL-4B affinity column with immobilised OmpC protein. The blue line shows the protein concentration measured in the nano-drop apparatus, and the red colour indicates the absorbance of the antibody elution solution. Elution with: **(A)** PBS excess unbound material, **(B)** PBS, **(C)** 1 M sodium chloride solution, **(D)** PBS, **(E)** 3 M potassium thiocyanate (KSCN), **(F)** PBS.

Specific anti-OmpC antibodies were obtained by affinity chromatography. Sepharose CL-4B with immobilised OmpC protein was used as the chromatography bed. Immobilisation was performed using CNBr activation of hydroxyl groups of Sepharose and further binding of protein to the activated chromatography bed. Human or mouse peripheral blood serum with high reactivity with bacterial OmpC protein was selected for specific anti-OmpC antibodies isolation (**Figure 3**). Affinity chromatography was performed on prepared OmpC-Sepharose CL-4B, and fractions with particular antibodies were concentrated, suspended in glycerol/PBS (1:1) solution and stored at -70°C.

For a single isolation of antibodies, the preparation using 4 ml of Sepharose bed CL-4B-OmpC and 500 µl of human serum (1:1 in PBS) yielded 600 µl of anti-OmpC antibodies (1:1 PBS: glycerol) at a concentration of 355 µg/ml (213 µg). Similarly, for a single isolation from mouse serum, 277 µg/ml (166 µg) of anti-OmpC antibodies were obtained.

### Reactivity and titres of human anti-OmpC antibodies with selected antigens

The most common deficiencies among PID are those associated with abnormalities in antibody production. They can be diagnosed by serological tests based on determining level of antibodies in human serum. Using OmpC protein, BSA-linear peptide conjugate and BSA-cyclic peptide conjugate as antigens in an ELISA assay, the levels of IgA and IgG class anti-OmpC antibodies were determined in the sera of healthy donors, patients with humoral primary immunodeficiencies (PID) and patients with recurrent upper respiratory tract infections (RRTI). In addition, comparing the antibodies’ reactivity with purified OmpC protein or two conjugates was intended to clarify whether the reactivity is determined by differences in the spatial conformation of the peptides used for conjugation and can affect the presentation of the antibody-binding epitope, thus affecting the antibody level in human serum.

#### Determination of anti-OmpC antibody titres of IgA class by ELISA

The epitope of the OmpC protein (GLNRYDERYIGC), recognised by human serum antibodies, is present in loop 3 of this protein, and its shortest antibody-reactive sequence is RYDERY. For the determination of anti-OmpC antibodies level an enzymatic assay was performed in which the titration plates were coated with OmpC protein (0.5 μg/100 μl), BSA-linear peptide (GLNRYDERYIGC) conjugate and BSA-cyclic peptide **[**GGLNRYDERYIG]C) conjugate, respectively.

The amount of antigen was calculated according to the determined number of epitopes per molecule of that antigen (OmpC – 1, BSA-linear peptide – 6, BSA-cyclic peptide – 4) and molecular mass (38 kDa, 75.204 kDa and 73.560 kDa, respectively) to use the same number of epitopes capable to bind anti-OmpC antibodies per well (0.5 µg, 0.167 µg and 0.25µg, respectively). That approach made it possible to show the difference in serum anti-OmpC antibodies titre between different groups of subjects.

To exclude non-specific interactions of serum components, a control well without an antigen was used. Blocking with BSA protein allowed for the exclusion of non-specific interactions between serum proteins and the carrier protein (BSA) used to obtain the conjugates. Interestingly, particularly high levels of nonspecific interactions were obtained for sera of patients with general inflammation (high CRP levels) (20, 21).

**Figure 4:**
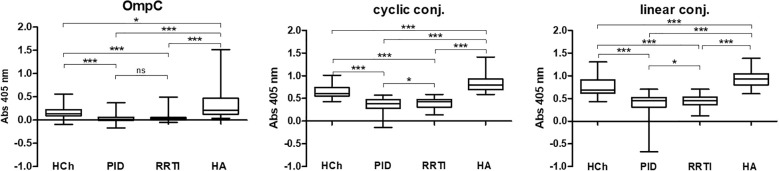
IgA class anti-OmpC antibody titres measured in sera of patients and healthy controls, tested using whole OmpC protein, cyclic peptide- or linear peptide-BSA conjugates, (statistical significance “P”: P < 0.05*, P < 0.001***). HCh, healthy children; PID, patients with primary immunodeficiency; RRTI, patients with recurrent respiratory tract infections; HA, healthy adults.

The statistical analysis of the titres of IgA class anti-OmpC antibodies in the sera of the different patient groups and control groups is shown in **Figure 4** and **Table 2**. It should be noted that IgG class antibodies are not as good marker as IgA for humoral deficiency determination (12), thus IgA antibodies were the major subject of the study.

The highest anti-RYDERY IgA antibody titres in sera are found in blood donors (healthy adults), lower in children without known immune disorders, and the lowest levels were observed among children diagnosed with PID/RRTI. These results also confirm our earlier results published in FEMS 2006 (12), where only OmpC protein was used as an antigen in ELISA test, to determine antibody titres in sera of smaller population of patients.

According to the results shown in **Table 2**, the highest reactivity with specific serum IgA was observed when a linear peptide conjugated with BSA was used as an antigen in the ELISA test (mean for sera of healthy children: 0.7659 ± 0.0319, for sera of children with PID: 0.3693 ± 0.0236, for sera of blood donors: 0.9437 ± 0.0230). Slightly lower reactivity was found when a cyclic peptide conjugated with BSA was used as an antigen in the ELISA (mean for sera of healthy children: 0.6523 ± 0.0222, for sera of children with PID: 0.3455 ± 0.0151, for sera of blood donors: 0.8403 ± 0.0215). The lowest reactivity was observed when the OmpC protein was used as antigen (mean for sera of healthy children: 0.1668 ± 0.0218, for sera of children with PID: 0.0393 ± 0.0077, for sera of blood donors: 0.3029 ± 0.0306) (**Table 2****).** Nevertheless, the most distinct differences between the groups were obtained in the test where the OmpC protein was used as an antigen.

**Table 2:** Averaged titres of IgA class antibodies, determined by ELISA to three antigens (OmpC protein, cyclic peptide- or linear peptide-BSA conjugate), and present in the sera of the patients (PID, RRTI) and control groups (healthy adults, healthy children).

Groups	Number of tested sera	Age of subjects [years] ± SD	IgA OmpC protein ± SD	IgA cyclic peptide conjugated with BSA ± SD	IgA linear peptide conjugated with BSA ± SD
Healthy children	40	10.6 ± 4.1	0.1668 ± 0.0218	0.6523 ± 0.0222	0.7659 ± 0.0319
PID patients	127	5.7 ± 4.4	0.0393 ± 0.0077	0.3455 ± 0.0151	0.3693 ± 0.0236
RRTI patients	83		0.0551 ± 0.0118	0.3967 ± 0.0115	0.4449 ± 0.0139
Healthy adults	66	30.2 ± 12.4	0.3029 ± 0.0306	0.8403 ± 0.0215	0.9437 ± 0.0230

SD, standard deviation. PID, primary immunodeficiency; RRTI, recurrent respiratory tract infections.

**Figure 5:**
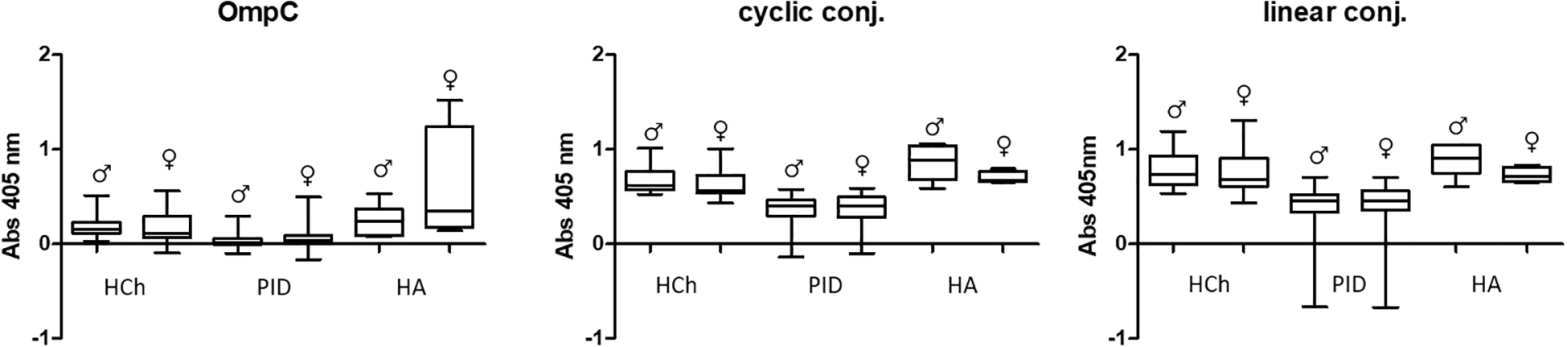
Comparative analysis of specific IgA class antibody titres in sera of women (♀) and men (♂) tested using OmpC protein, linear peptide- or cyclic peptide-BSA conjugate used for ELISA. HCh, healthy children; PID, patients with primary immunodeficiency; HA, healthy adults.

Researchers working on this topic have noted that antibody levels may vary between men and women (22–24). The statistical analysis of the results (**Figure 5** and **Table 3**) revealed that some differences in IgA levels were observed when using the OmpC protein; however, no distinct differences were obtained in the tests with both conjugates.

The most significant differences regarding the level of specific IgA antibodies in the sera of the subjects were observed in the group of adult blood donors. However, the difference was not statistically significant, due to a limited number of samples tested (**Figure 5**) (**Table 3**). In the other groups (healthy children and patients with PID), statistically significant differences in antibody levels were obtained only for females and males from the group of PID patients designated for the OmpC as an antigen. The above data show that the level of specific anti-OmpC antibodies of the IgA class, determined in the sera of the subjects, does not correlate with gender when using conjugates as antigens.

**Table 3:** Averaged titres of specific IgA class antibodies, determined in the sera of the study groups against the three antigens used in the ELISA (OmpC protein, cyclic peptide conjugated with BSA, linear peptide conjugated with BSA) analysed by gender.

Groups	Sex	Number of tested sera	IgA anti-OmpC ± SD	IgA anti-cyclic peptide conjugated with BSA ± SD	IgA anti-linear peptide conjugated with BSA ± SD
Healthy children	M	17	0.1732 ± 0.0266	0.6732 ± 0.0311	0.7760 ± 0.0461
	W	23	0.1621 ± 0.0329	0.6368 ± 0.0313	0.7584 ± 0.0446
PID patients	M	117	0.0247 ± 0.0058	0.3635 ± 0.0137	0.3924 ± 0.0205
	W	81	0.0658 ± 0.0119	0.3679 ± 0.0175	0.4071 ± 0.0267
Healthy adults	M	7	0.2429 ± 0.0617	0.8467 ± 0.0696	0.8699 ± 0.0618
	W	4	0.5873 ± 0.3153	0.6968 ± 0.0327	0.7265 ± 0.0419

SD, standard deviation; M, men; W, woman. PID, primary immunodeficiency.

**Figure 6:**
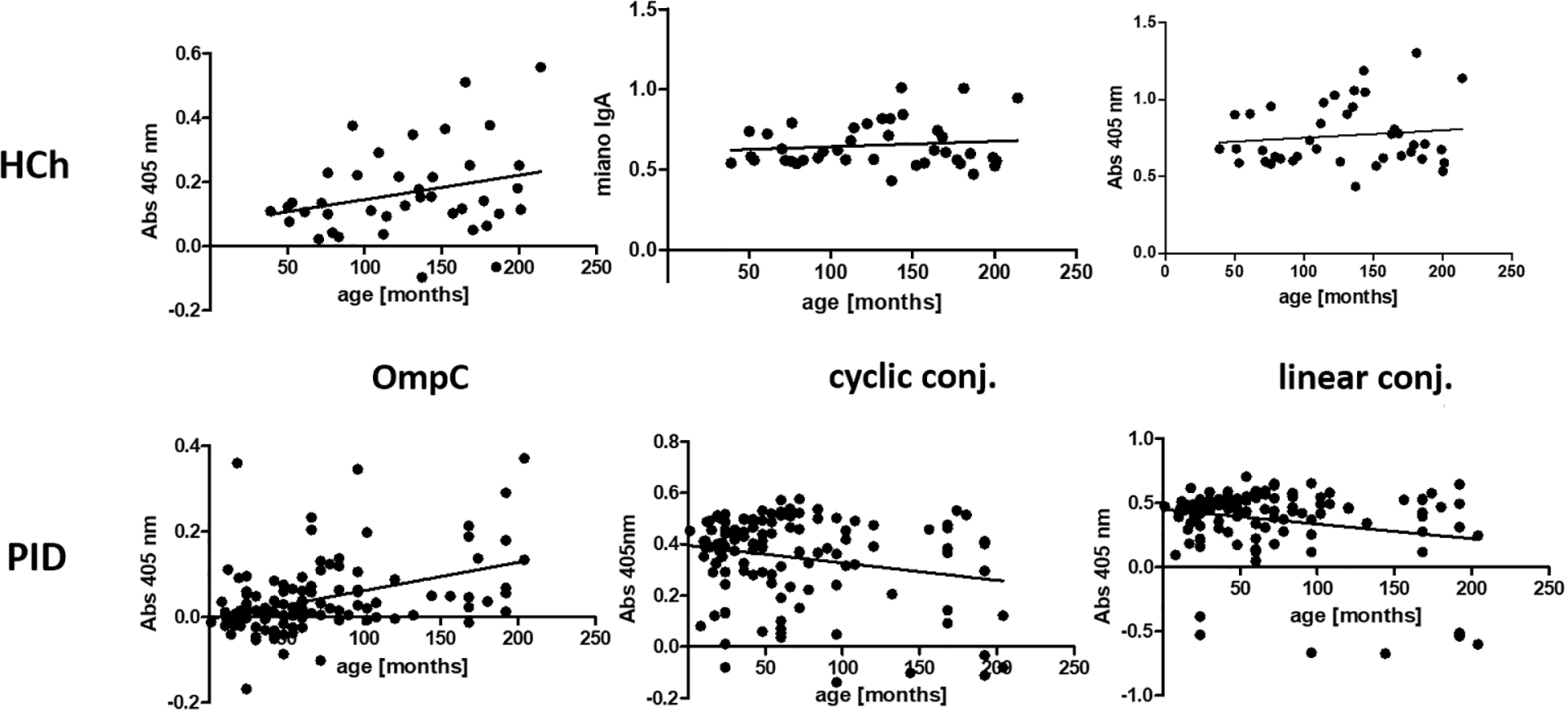
Presentation of the titres of specific IgA class antibodies in the sera of the healthy children and PID patients study groups, determined for the three antigens used in the ELISA (OmpC protein, linear peptide conjugated with BSA, cyclic peptide conjugated with BSA), according to the age of the subjects. HCh, healthy children; PID, patients with primary immunodeficiency.

We have also performed a statistical analysis to determine whether the titre of IgA is age-dependent (**Figure 6**, **Table 4**).

**Table 4:** Presentation of the values of r-Pearson correlation coefficients from the analysis of the dependence of specific IgA class antibody titres on the age of healthy children or PID patients (weak correlation r from -0.5 to 0.0 and 0.0 to 0.5, strong correlation r from -1.0 to -0.5 and 0.5 to 1.0).

IgA	Number of tested sera	Antigen used to the test	Coefficient r-Pearson	P-value (level of statistical significance)
Healthy children	40	OmpC protein	0,273	0,088
		cyclic peptide conjugated with BSA	0,120	0,463
		linear peptide conjugated with BSA	0,121	0,458
PID patients	119	OmpC protein	0,416	< 0,001 ***
		cyclic peptide conjugated with BSA	-0,212	0,021 *
		linear peptide conjugated with BSA	-0,224	0,014 *

A P value < 0.05 indicates statistical significance (P < 0.05*, P < 0.01**, P < 0.001***). PID, primary immunodeficiency.

Analysis of the r-Pearson correlation for the determined level of specific IgA class antibodies in the sera of healthy children indicates an increasing trend in antibody titres with the child’s age (**Table 4**). However, correlation coefficient r values less than 0.5 indicate a weak correlation. The level of statistical significance for healthy children is greater than 0.05, which does not allow us to reject the null hypothesis (H0 - antibody titres in children do not depend on their age). The results obtained for the sera of healthy children do not show a strong correlation between the level of anti-OmpC antibodies of the IgA class. Patients over the age of 18 were excluded from the analysis due to the lack of detailed data on their age. Among the obtained results of specific antibody titres in sera from healthy children (without immunodeficiencies) are those for sera from children over 3 years old. An initial decrease in the titres of antibodies transferred across the placenta from the mother was not observed. The expected initial reduction in antibody titres among children under 3 years of age is due to the gradual loss of antibodies transferred to children via the placenta from the mother.

The titre of IgA class antibodies in the sera of PID patients determined against the OmpC protein indicates that its level increases with the age of the children. The r-Pearson correlation coefficient is 0.416 (r < 0.5), indicating a low correlation. However, p < 0.001 suggests a high statistical significance for the obtained result. For the level of specific antibodies in the sera of PID patients determined against cyclic peptide and linear peptide conjugated with BSA, negative results were obtained at -0.212 and - 0.224, respectively. These results suggest a decrease in the observed titres of anti-OmpC antibodies with age in the sera of immunodeficient children. The P-values are 0.021 when using cyclic peptide conjugated with BSA as an antigen, and 0.014 (P < 0.05) against linear peptide conjugated with BSA, respectively, what confirms that the antibody titre is age dependent.

#### Determination of IgG class antibody titres by ELISA

IgG class antibodies are an element of the so-called long-term memory and their presence indicates that the patient has been in contact with a specified pathogen. Patients with humoral immunodeficiencies, often do not produce this class of antibodies (or its subclasses), or these antibodies are non-functional, as are IgA class antibodies. Such patients are at risk of significantly more frequent recurrent infections, and their course is much more severe than in patients without immune burdens. Determination of and comparison of antibody titres of this class in patients with PID and patients without immunodeficiencies, is essential for the development of a serological test for diagnosis of deficiencies.

**Figure 7:**
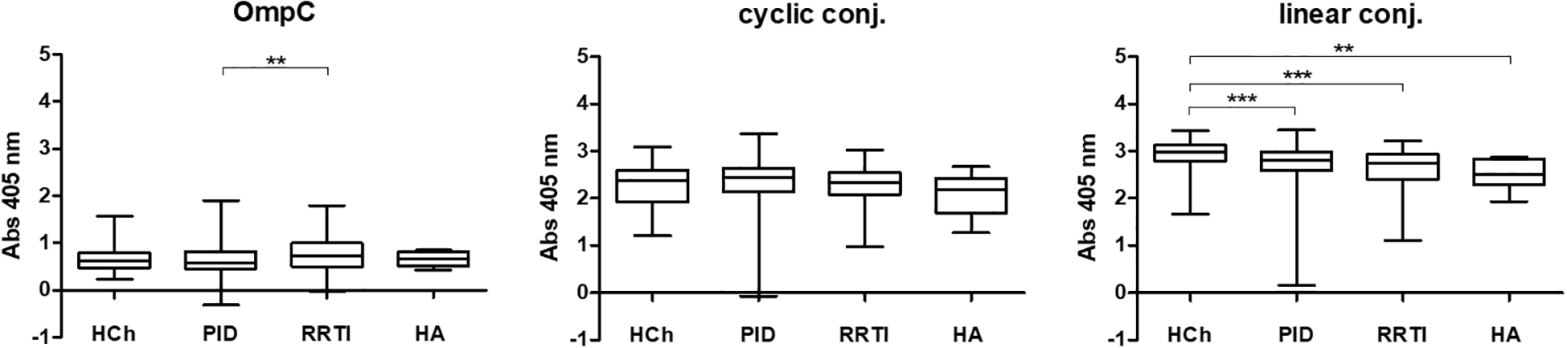
IgG class anti-OmpC antibody titres determined in sera of patients and healthy controls, tested using whole OmpC protein, cyclic peptide- or linear peptide-BSA conjugate, (statistical significance “P”: P < 0.01**, P < 0.001***). HCh, healthy children; PID, patients with primary immunodeficiency; RRTI, patients with recurrent respiratory tract infections, HA, healthy adults.

**Figure 8:**
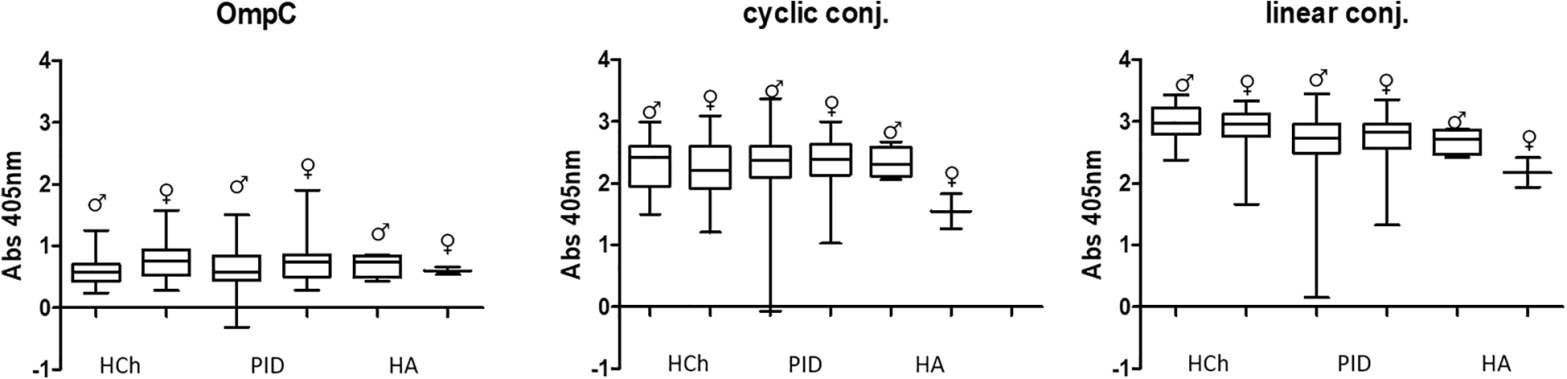
Comparative analysis of specific IgG class antibody titres in the sera of women (♀) and men (♂) tested using OmpC protein, linear peptide- or cyclic peptide-BSA conjugate used for ELISA. HCh, healthy children; PID, patients with primary immunodeficiency; HA, healthy adults.

In all the tested groups, the average level of antibodies determined against the OmpC protein is significantly lower than that determined when any of the conjugates is used. In addition, we also observed that the linear peptide conjugated with BSA bound antibodies more efficiently than the cyclic peptide conjugated with BSA. There was no significant difference in IgG antibody patterns between healthy children and PID/RRTI groups, indicating the lack of their diagnostic potential (**Figure 7**, **Figure 8**, **Tables 5**–**7**).

**Table 5:** Averaged titres of specific IgG class antibodies in the sera of the patient and control groups, determined against the three antigens used in the ELISA (OmpC protein, linear peptide conjugated with BSA, cyclic peptide conjugated with BSA).

	Number of tested sera	IgG anti-OmpC protein ± SD	IgG anti-cyclic peptide conjugated with BSA ± SD	IgG anti-linear peptide conjugated with BSA ± SD
Healthy children	37	0.6722 ± 0.0481	2.2660 ± 0.0758	2.9270 ± 0.0557
PID patients	117	0.6482 ± 0.0276	2.3410 ± 0.0502	2.7300 ± 0.0454
RRTI patients	76	0.7878 ± 0.0410	2.2630 ± 0.0522	2.6420 ± 0.0473
Healthy adults	6	0.6625 ± 0.0649	2.0760 ± 0.1987	2.5110 ± 0.1396

SD, standard deviation. PID, primary immunodeficiency; RRTI, recurrent respiratory tract infections.groups.

**Table 6:** Summary of the averaged titre of specific antibodies of the IgG class, determined against three antigens (OmpC protein, linear peptide conjugated with BSA, cyclic peptide conjugated with BSA) determined in the sera of the study groups, taking into account the number and sex of the subjects.

Groups	Sex	Number of studied sera	IgG anti-OmpC protein ± SD	IgG anti-cyclic peptide conjugated with BSA ± SD	IgG anti-linear peptide conjugated with BSA ± SD
Healthy children	M	16	0.6029 ± 0.0682	2.2960 ± 0.1059	2.9740 ± 0.0723
	W	21	0.7250 ± 0.0661	2.2430 ± 0.0184	2.8920 ± 0.0818
PID patients	M	106	0.6438 ± 0.0302	2.2830 ± 0.0554	2.6510 ± 0.0508
	W	76	0.7509 ± 0.0363	2.3430 ± 0.0473	2.7450 ± 0.0453
Healthy adults	M	4	0.6933 ± 0.0946	2.3390 ± 0.1263	2.6800 ± 0.1034
	W	2	0.6010 ± 0.0610	1.5490 ± 0.2830	2.1750 ± 0.2405

SD, standard deviation; M, men; W, woman. PID, primary immunodeficiency.

**Table 7:** Presentation of a P value indicating statistical significance.

IgG	Anti-OmpC protein			Anti-cyclic peptide conjugated with BSA			Anti-linear peptide conjugated with BSA		
	Healthy children	PID patients	Healthy adults	Healthy children	PID patients	Healthy adults	Healthy children	PID patients	Healthy adults
Men/women	0.2137	0.0240 *	0.5638	0.7361	0.4362	0.0370 *	0.4737	0.1853	0.0775

Result P < 0.05 is a statistically significant result (P < 0.05*).

**Figure 9:**
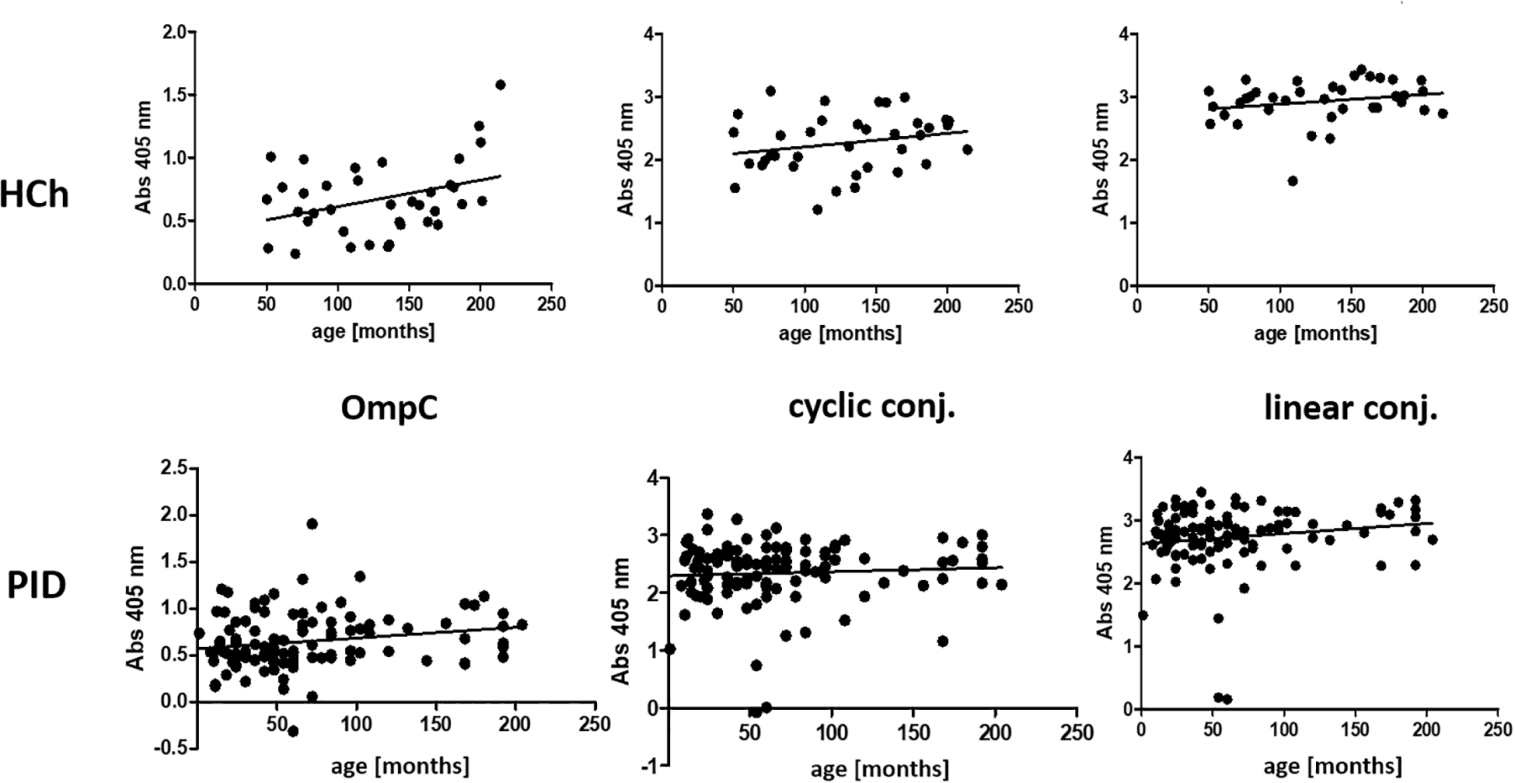
Presentation of the average titre of specific IgG class antibodies in the sera of the healthy children and PID patients study groups, determined for the three antigens used in the ELISA (OmpC protein, linear peptide conjugated with BSA, cyclic peptide conjugated with BSA), according to the age of the subjects. HCh, healthy children; PID, patients with primary immunodeficiency.

Analysing the levels of specific IgG-class anti-OmpC antibodies in the study groups according to age, no significant differences were observed, as evidenced by an r-Pearson coefficient value of less than 0.5 and a P value > 0.05 (**Figure 9**) (**Table 8**).

**Table 8:** Summary values of r-Pearson correlation coefficients from the analysis of the dependence of specific IgG class antibody titres, determined for three antigens (OmpC protein, linear peptide conjugated with BSA, cyclic peptide conjugated with BSA), on the age of patients (weak correlation r from -0.5 to 0.0 and 0.0 to 0.5, strong correlation r from -1.0 to -0.5 and 0.5 to 1.0).

IgG	Number of studied sera	Antigen used in ELISA test	Coefficient r-Pearson	P-value (level of statistical significance)
Healthy children	37	OmpC protein	0.351	0.033
		cyclic peptide conjugated with BSA	0.227	0.176
		linear peptide conjugated with BSA	0.218	0.194
PID patients	111	OmpC protein	0.195	0.041
		cyclic peptide conjugated with BSA	0.065	0.496
		linear peptide conjugated with BSA	0.164	0.086

PID, primary immunodeficiency.

Given the increasing antibiotic resistance in bacteria, this also justifies the research conducted on developing an effective therapeutic in the form of specific anti-OmpC antibodies for supplementing antibody deficiencies in patients with PID.

### Studies conducted on an animal model

In our previous studies, we investigated the potential of using OMP protein vaccination to provide protection against *S. flexneri* 3a infection. An analysis of the protective properties of native and recombinant OmpC proteins was performed on a mouse model using an infection with an LD_100_ dose of *S. flexneri* 3a, and the results were recently published in (2). In the present work, we aimed to investigate whether isolated, specific anti-OmpC antibodies can have protective activity against *S. flexneri* infection, which could be used in the future to supplement antibody deficiencies in patients with PID. The studies were conducted on an animal model to confirm the protective effect of anti-OmpC antibodies (isolated from mice and human serum) against infections caused by *S. flexneri* bacilli (**Table 9**).

The LD_50_ dose of *S. flexneri* 3a for BALB/c mice was determined based on the results of animal model studies conducted earlier in our laboratory (15). The procedure was performed on forty 6–9 week-old female mice of the BALB/c strain. Each of the four groups of animals consisted of 10 individuals. The animals were immunised three times at 24-hour intervals.

After administration of antibody preparations, the animals were healthy and showed no signs of disease. In the model experiment conducted, it was found that 24 hours after infection, four mice from group No. 1 (the control group) died, and the remaining mice exhibited signs of infection, including a dull coat, lethargy, and impaired response to stimuli.

Animals in groups No. 1 and No. 2 had more severe symptoms than those in groups No. 3 and No. 4. Another two mice in the first group died 48 hours after infection. In the remaining animals, the disease symptoms decreased; however, those in groups No. 1 and No. 2 were more severe than those in groups No. 3 and No. 4. At 72 hours after infection, the animals appeared significantly improved compared to previous days. Group No. 1 seemed to be slightly worse than the other groups of animals. The mortality rate of the control group (mice immunised with PBS solution) was 60% (6/10 animals died) over the course of the experiment, i.e. 72 hours after pathogen administration.

The remaining animals survived, which clearly confirms the protective effect of the administered antibodies. The expected mortality of animals in the control group was 50% (LD_50_ - Lethal Dose 50%), but the experimentally determined mortality was 60%. This difference is most likely due to individual differences between the animals. Animals in group 2, immunised thrice with 50 µg doses of human antibodies, as well as animals in group 4, immunised thrice with 50 µg doses of mouse antibodies, showed stronger signs of infection than animals in group 3, which received a higher dose of human antibodies (3×100 µg) three times. The experiment clearly confirmed the protective effect of anti-OmpC antibodies against the development of shigellosis. The results obtained are the basis for further work on antibody-supplemented therapeutics in patients with humoral immunodeficiencies.

## Discussion

OmpC protein is a component of the outer membrane of *S. flexneri* 3a bacilli and a β-barrel porin whose fragments (loops) are exposed on the outside of bacterial cell. It belongs to the so-called major proteins of *S. flexneri* and, compared to other outer membrane proteins, shows the highest immunoreactivity with human cord blood serum antibodies (12). This protein, as other proteins from the class of OMP family (25, 26), due to the exposure of its fragments to the outside of the bacterial membrane, is recognised by the host immune system, thanks to which it is the main antigen involved in the induction of the immune response, which was confirmed in protection experiments on a mouse model (27, 28).

Preliminary studies on the presence of anti-OmpC antibodies in human serum have demonstrated their presence in the serum of healthy individuals, and absent or at significantly lower levels in the serum of immunodeficient children (12). Anti-OmpC antibodies have also been found in human cord blood serum (15). The anti-OmpC antibodies found in cord blood are passed from the mother to the child, which indicates that they are protective in nature.

Immunoglobulin A (IgA) is involved in the immune defence of respiratory, gastrointestinal, and genitourinary mucous membranes. Its serum level is the second highest of all immunoglobulins after immunoglobulin G (IgG). IgA is mainly involved in neutralising viruses, binding toxins, agglutinating bacteria, and preventing them from binding to epithelial cells in the mucous membranes (29).

Selective IgA deficiency is the most common primary immunodeficiency, but remains undiagnosed and in most cases, as the vast majority of people with IgA deficiency do not have disease symptoms (30). The maturation trajectory of IgA synthesis is slow and can persist even after age 12; the proper diagnosis of selective IgA deficiency (SIgAD) can be based on both specific and probable symptoms (31). Although most patients with SIgAD do not develop symptoms, some conditions related to SIgAD have been reported, particularly recurrent respiratory tract infections and infections caused by encapsulated microorganisms (29). Due to the difficulties of correct and effective diagnosis of patients, the first diagnosis is usually made at the age of 5 years and older, after a history of multiple infections (from 5 to even more than 20 hospitalizations until a correct diagnosis is made, due to sinusitis, bronchitis, pneumonia, otitis, diarrhoea, malabsorption, sepsis and other) (32). Although sinopulmonary infections with encapsulated bacteria are the most common infections, primary antibody deficiencies may also occur with infections caused by other usual or unusual microorganisms affecting various organs (33). Currently, there is no standard treatment for selective IgA deficiency. The therapeutic approach is individualised according to the patient’s clinical status (34). Early diagnosis and timely initiation of immunoglobulin replacement therapy (IgRT) can be of great benefit to the patient. Nevertheless, the diagnosis is challenging, and based on Ig blood tests. Humoral response testing, on the other hand, requires vaccination with either tetanus toxoid or diphtheria toxin and is considered a highly complex diagnostic procedure (35, 36).

An intensive research is now ongoing into finding new PID diagnostic methods which are based on classical and genetic methods (43–45). Additionally, IgA replacement therapy carries the risk of anaphylactic shock following the administration of the immunoglobulin preparation.

In the present work, we analysed the reactivity and the level of specific anti-OmpC *S. flexneri* 3a antibodies in a large number of sera from healthy individuals and immunocompromised patients. Additionally, we tested the protective properties of isolated anti-OmpC antibodies.

During the experiments, the OmpC protein isolated from *S. flexneri* 3a using valeric acid and further purified by gel filtration and ion-exchange chromatography was used. Additionally, two synthetic peptides containing the RYDERY sequence in two forms – linear and cyclic – were utilised. The cyclic peptide was considered to mimic the natural spatial arrangement of the binding epitope.

The immunoblotting experiment proved that isolated OmpC maintained its reactivity with human serum containing specific anti-OmpC IgG (**Figure 1**). Our previous results showed that an epitope of the OmpC protein from *S. flexneri* 3a, with the sequence GLNRYDERYIGC, had the highest immunogenicity and was responsible for the binding of human anti-OmpC antibodies (15, 19). For the immunoreactivity analyses, we have conjugated RYDERY epitope peptides with BSA monomer, chromatographically purified from BSA dimers. The degree of loading of the carrier protein with peptides, determined using MALDI-TOF, was around 6 for the BSA/linear peptide conjugate and 4 for BSA/cyclic with some variable degree of peptide occupancy of the carrier protein (**Figure 2**). As the loop of the cyclic peptide occupies more space around the carrier protein, not all available amino groups on the BSA molecule, could attach peptides due to the steric hindrance. The linear peptide, which occupies less space around the BSA molecule, was able to saturate this carrier protein to a greater extent than cyclic peptides.

The obtained results showed that for OmpC and both conjugates IgA titres were significantly lower for PID and RRTI patients in comparison with healthy controls (**Figure 4**). The IgG antibody titres did not differ significantly between patients and controls (**Figure 7**, **Table 7**).

Several publications concerning this topic suggest that the level of anti-OmpC antibodies may differ between men and women (22–24). Our results have shown that, surprisingly, the levels of specific IgA antibodies differ significantly between men and women in the groups of PID patient and healthy adult groups when using native OmpC protein (**Figure 5**). However, there is no significant gender difference in IgA levels in PID patients, and much less difference in healthy adults when using conjugates as antigen. For IgG analysis, differences of anti-OmpC antibody titres were visible mostly in analysis with cyclic conjugate (**Figure 8**, **Table 6**). These findings may suggest advantages of using of the linear conjugate in addition to the whole OmpC protein in immunochemical assays.

The analysis of results indicates that, in contrast to healthy individuals, the level of IgA in patients’ sera is overall lower when using the whole OmpC protein for analysis, there was no observable decrease in IgA levels. It is well known that in the first months after birth, the antibodies are transferred from the mother to the newborn. The level of maternal antibodies in an infant’s serum decreases over an average of 6–12 months in the infant’s bloodstream. The level of antibodies produced by the child’s body gradually increases; however, this was not observed for IgA in the sera of patients with PID.

The differences in reactivity obtained may be due to several reasons, but the most obvious appear to be differences in epitope exposure. OmpC protein has only one binding epitope in its structure, and during immobilisation of this protein to the titration plate surface, the epitope may be partially inaccessible to antibodies, resulting in a low reactivity signal. Whereas peptides conjugated with BSA on their surface contain several epitopes, so in each conjugate arrangement, a portion of the epitopes is exposed and accessible to antibodies, respectively. This explains the significantly higher reactivity of both conjugates compared to the OmpC protein. The average occupancy of the BSA monomer is 6 for linear peptide and 4 for cyclic peptide, so that the linear conjugate showed greater availability of epitopes and thus higher reactivity. Both conjugates exhibited high reactivity with sera. The age-dependent increase in antibody titre, observed in experiments with whole OmpC, may be also due to the presence of various other epitopes on the OmpC molecule’s surface, which react with the vast array of antibodies in serum against different antigens. Apparently, even children with PID acquire gradual immunity after contact with other bacteria, but for a particular epitope of RYDERY, the level of antibodies shows a noticeable difference between groups. Therefore, the specific epitope, rather than the whole OmpC protein, must be used in such immunological testing; consequently, the test should be constructed based on a protein-peptide conjugate. As RRTI patients are always suspected of having PID, the conclusive diagnosis must be made. The proposed testing method, using the BSA-peptide conjugate with the RYDERY sequence, may serve as a simpler alternative to complex diagnostic schemes that involve patient vaccination and testing the antibody response to immunisation.

The current results regarding the level of IgG anti-OmpC in sera from immunodeficient patients differ slightly from those published in our earlier 2006 study (12). This may be because the earlier study was conducted on a much smaller number of sera from immunodeficient patients. Additionally, in that work, we utilised the OmpC protein as an antigen to determine antibody levels in an ELISA, which was sufficient for screening purposes. Conjugates with selected epitopes will be the better choice for constructing a test for the early detection of immunoglobulin deficiencies.

To test the immunoprotective potential of anti-OmpC antibodies, we performed experiments using a mouse model. Specific anti-OmpC antibodies from human or mouse serum preparations were obtained by affinity chromatography. The preparations were tested to determine whether the antibodies protect animals from developing *Shigella* infection. Both antibodies of human and mouse origin had similar protective activity (defined as the animal survival rate in the experiment conditions after the peritoneal administration of LD_50_ bacterial dose) against *Shigella* infection in mouse model (**Table 9**). This leads us to believe that both doses of human antibodies protected the animals from the development of infection and death, and that the higher total dose of specific human antibodies (3×100 µg) anti-OmpC protected the animals more effectively, as evidenced by the much milder signs of infection in this group. Animals immunised with a lower dose of human or mouse antibodies (3×50 µg Ab) survived the procedure, but showed different symptoms during infection. Animals treated with mouse antibodies had milder symptoms compared to those treated with human antibodies. This indicates that the animals’ immune systems acted more effectively against a pathogen that had been co-opsonised with their own species’ antibodies.

**Table 9:** Summary of results obtained after passive immunisation of mice with preparations of human or mouse anti-OmpC *S. flexneri* 3a antibodies and application of the LD_50_ dose of bacteria.

Groups	Day 1	Day 2	Day 3	Number of dead animals, after [hour] pathogen administration				% Protection
				24 hr	48 hr	72 hr	Σ	
1	PBS (control)	PBS	PBS (control)	4	2	0	6	40
		1 hour interval						
		LD_50_ *Shigella flexneri* 3a						
2	50 µg Ab (human)	50 µg Ab (human)	50 µg Ab (human)	0	0	0	0	100
		1 hour interval						
		LD_50_ *Shigella flexneri* 3a						
3	100 µg Ab (human)	100 µg Ab (human)	100 µg Ab (human)	0	0	0	0	100
		1 hour interval						
		LD_50_ *Shigella flexneri* 3a						
4	50 µg Ab (mouse)	50 µg Ab (mouse)	50 µg Ab (mouse)	0	0	0	0	100
		1 hour interval						
		LD_50_ *Shigella flexneri* 3a						

There were 10 animals in each group.

Anti-OmpC *S. flexneri* 3a antibodies are known to react with other proteins extracted from *Enterobacteriaceae* strains (37). Due to the high homology of *Enterobacteriaceae* proteins, anti-OmpC *S. flexneri* antibodies are expected to confer cross-protection against infections caused by other strains of this family. Our previous studies in a mouse model showed protection of animals immunised with protein preparations from *S. flexneri* 3a after infection with different strains of *Enterobacteriaceae* (27, 38). Wang et al. (39) showed that mouse serum after inoculation with recombinant OmpC protein shows high reactivity with proteins of many other bacterial species such as *E. coli*, *S. flexneri* and *S. dysenteriae*, while with representatives of other Gram-negative bacterial species such as *Pseudomonas aeruginosa*, the serum shows no reactivity (39).

Confirmation of the protective properties of anti-OmpC antibodies allows the development of an antibody-deficient supplement formulation. In addition, due to the existing homology of the proteins of major *Enterobacteriaceae*, the serum of animals immunised with OmpC protein from *S. flexneri* 3a, was reactive with proteins of other *Shigella* species and other *Enterobacteriaceae* (12, 40, 41). The cross-reactivity of anti-*Shigella* antibodies with other bacteria broadens the range of action of the formulation and increases its appeal as a potential drug. The high degree of homology of the structures of the main proteins of the outer membrane of *Enterobacteriaceae* family bacilli allows us to believe that the administration of anti-OmpC antibodies to *S. flexneri* will protect patients with PIDs from the development of infection not only with *Shigella* bacilli, but also provide them with protection against the development of infection caused by other representatives of this group of bacteria. In conclusion, the results of the presented studies demonstrate that the use of the cyclic/linear peptide-BSA conjugate offers a significant advantage over the use of the complete OmpC protein in terms of reactivity with human and mouse serum antibodies. In addition to the absence of bacterial material in the assay and the lower cost of conjugate production, the method does not yield significant differences in IgA antibody levels depending on the patient’s gender. Both conjugates also clearly differentiate between healthy and sick patients in an age-dependent assay. Notably, for the conjugates, higher values are also obtained in the ELISA test, which reduces the error rate. The results of the animal experiments strongly indicate the possibility that specific anti-OmpC antibodies may be beneficial in the complementary therapy of patients with PIDS. We hope, that further research, followed by the appropriate clinical trials will result in this type of protocol being incorporated into clinical therapies. The promising results presented here show, that there is a need for further investigation using wider cohorts of samples, long term follow-up of patients involved in this study, and population not limited to one region of Poland only.

Primary immunodeficiencies are a pioneering field of medicine, where there is still much to be explored. Discovering new, cutting-edge treatments is a constant necessity as we uncover new types of diseases and gain a deeper understanding of their patterns.

## Data Availability

The raw data supporting the conclusions of this article will be made available by the authors, without undue reservation.
